# The Burden of *Cryptosporidium* Diarrheal Disease among Children < 24 Months of Age in Moderate/High Mortality Regions of Sub-Saharan Africa and South Asia, Utilizing Data from the Global Enteric Multicenter Study (GEMS)

**DOI:** 10.1371/journal.pntd.0004729

**Published:** 2016-05-24

**Authors:** Samba O. Sow, Khitam Muhsen, Dilruba Nasrin, William C. Blackwelder, Yukun Wu, Tamer H. Farag, Sandra Panchalingam, Dipika Sur, Anita K. M. Zaidi, Abu S. G. Faruque, Debasish Saha, Richard Adegbola, Pedro L. Alonso, Robert F. Breiman, Quique Bassat, Boubou Tamboura, Doh Sanogo, Uma Onwuchekwa, Byomkesh Manna, Thandavarayan Ramamurthy, Suman Kanungo, Shahnawaz Ahmed, Shahida Qureshi, Farheen Quadri, Anowar Hossain, Sumon K. Das, Martin Antonio, M. Jahangir Hossain, Inacio Mandomando, Tacilta Nhampossa, Sozinho Acácio, Richard Omore, Joseph O. Oundo, John B. Ochieng, Eric D. Mintz, Ciara E. O’Reilly, Lynette Y. Berkeley, Sofie Livio, Sharon M. Tennant, Halvor Sommerfelt, James P. Nataro, Tomer Ziv-Baran, Roy M. Robins-Browne, Vladimir Mishcherkin, Jixian Zhang, Jie Liu, Eric R. Houpt, Karen L. Kotloff, Myron M. Levine

**Affiliations:** 1 Centre pour le Développement des Vaccins, Bamako, Mali; 2 Center for Vaccine Development, University of Maryland School of Medicine, Baltimore, Maryland, United States of America; 3 Department of Medicine, University of Maryland School of Medicine, Baltimore, Maryland, United States of America; 4 Emergent Biosolutions, Gaithersburg, Maryland, United States of America; 5 National Institute of Cholera and Enteric Diseases, Kolkata, India; 6 Department of Paediatrics and Child Health, the Aga Khan University, Karachi, Pakistan; 7 International Centre for Diarrhoeal Disease Research, Mohakhali, Dhaka, Bangladesh; 8 Medical Research Council (United Kingdom) Unit, Fajara, Gambia; 9 GSK Vaccines, Wavre, Belgium; 10 Centro de Investigação em Saúde da Manhiça, Maputo, Mozambique; 11 Instituto Nacional de Saúde, Ministério de Saúde, Maputo, Mozambique; 12 ISGlobal, Barcelona Ctr. Int. Health Res. (CRESIB), Hospital Clínic—Universitat de Barcelona, Barcelona, Spain; 13 Global Disease Detection Division, Kenya Office of the US Centers for Disease Control and Prevention, Nairobi, Kenya; 14 Kenya Medical Research Institute/Centers for Disease Control and Prevention, Kisumu, Kenya; 15 Division of Foodborne, Waterborne and Environmental Diseases, Centers for Disease Control and Prevention, Atlanta, Georgia, United States of America; 16 Centre of Intervention Science in Maternal and Child Health, Centre for International Health, University of Bergen, Bergen, and Department of International Public Health, Norwegian Institute of Public Health, Oslo, Norway; 17 Department of Epidemiology and Preventive Medicine, School of Public Health, Sackler Faculty of Medicine, Tel Aviv University, Ramat Aviv, Israel; 18 Department of Microbiology and Immunology, The University of Melbourne, Murdoch Children’s Research Institute, Royal Children's Hospital, Parkville, Victoria, Australia; 19 Division of Infectious Diseases and International Health, Department of Medicine, University of Virginia, Charlottesville, Virginia, United States of America; 20 Department of Pediatrics, University of Maryland School of Medicine, Baltimore, Maryland, United States of America; The Johns Hopkins University, UNITED STATES

## Abstract

**Background:**

The importance of *Cryptosporidium* as a pediatric enteropathogen in developing countries is recognized.

**Methods:**

Data from the Global Enteric Multicenter Study (GEMS), a 3-year, 7-site, case-control study of moderate-to-severe diarrhea (MSD) and GEMS-1A (1-year study of MSD and less-severe diarrhea [LSD]) were analyzed. Stools from 12,110 MSD and 3,174 LSD cases among children aged <60 months and from 21,527 randomly-selected controls matched by age, sex and community were immunoassay-tested for *Cryptosporidium*. Species of a subset of *Cryptosporidium*-positive specimens were identified by PCR; GP60 sequencing identified anthroponotic *C*. *parvum*. Combined annual *Cryptosporidium*-attributable diarrhea incidences among children aged <24 months for African and Asian GEMS sites were extrapolated to sub-Saharan Africa and South Asian regions to estimate region-wide MSD and LSD burdens. Attributable and excess mortality due to *Cryptosporidium* diarrhea were estimated.

**Findings:**

*Cryptosporidium* was significantly associated with MSD and LSD below age 24 months. Among *Cryptosporidium*-positive MSD cases, *C*. *hominis* was detected in 77.8% (95% CI, 73.0%-81.9%) and *C*. *parvum* in 9.9% (95% CI, 7.1%-13.6%); 92% of *C*. *parvum* tested were anthroponotic genotypes. Annual *Cryptosporidium*-attributable MSD incidence was 3.48 (95% CI, 2.27–4.67) and 3.18 (95% CI, 1.85–4.52) per 100 child-years in African and Asian infants, respectively, and 1.41 (95% CI, 0.73–2.08) and 1.36 (95% CI, 0.66–2.05) per 100 child-years in toddlers. Corresponding *Cryptosporidium*-attributable LSD incidences per 100 child-years were 2.52 (95% CI, 0.33–5.01) and 4.88 (95% CI, 0.82–8.92) in infants and 4.04 (95% CI, 0.56–7.51) and 4.71 (95% CI, 0.24–9.18) in toddlers. We estimate 2.9 and 4.7 million *Cryptosporidium*-attributable cases annually in children aged <24 months in the sub-Saharan Africa and India/Pakistan/Bangladesh/Nepal/Afghanistan regions, respectively, and ~202,000 *Cryptosporidium*-attributable deaths (regions combined). ~59,000 excess deaths occurred among *Cryptosporidium*-attributable diarrhea cases over expected if cases had been *Cryptosporidium*-negative.

**Conclusions:**

The enormous African/Asian *Cryptosporidium* disease burden warrants investments to develop vaccines, diagnostics and therapies.

## Author Summary

*Cryptosporidium* is a protozoan that causes diarrhea and malnutrition in young children in developing countries, and is associated with diarrhea cases and outbreaks in developed countries. To date, limited information exists on the burden of *Cryptosporidium* diarrheal disease in sub-Saharan Africa and South Asia, where most diarrheal disease deaths occur. We estimated the burden of *Cryptosporidium*-diarrhea and associated deaths in these regions using data from the Global Enteric Multicenter Study (GEMS). *Cryptosporidium* was associated with diarrhea mainly in children aged <24 months. Infections began in the first few months of life but clinical episodes of *Cryptosporidium*-associated diarrhea illness peaked at age 6–11 months. The annual number of *Cryptosporidium*-attributable diarrhea episodes was estimated at 2.9 and 4.7 million in children aged <24 months in sub-Saharan Africa and in the India/Pakistan/Bangladesh/Afghanistan/Nepal region of South Asia, respectively. In both regions combined, *Cryptosporidium* is estimated to contribute to approximately 202,000 deaths per year, and to ~59,000 more deaths in *Cryptosporidium*-attributable cases than if those cases had been negative for *Cryptosporidium*. Our study highlights the enormous burden attributable to *Cryptosporidium* in Africa and Asia, which underscores the need for developing vaccines and treatments to reduce this burden.

## Introduction

*Cryptosporidium*, the highly infectious protozoan that causes diarrhea in immunocompetent and immunocompromised subjects [1–4], is transmitted via contaminated water or food [1,3,5], swimming or bathing in surface waters [1,3] and by direct person-to-person contact [6], particularly in developing country settings of suboptimal sanitation and limited access to safe drinking water [1,3,4]. Clinical cryptosporidiosis ranges from self-limited mild diarrhea (most commonly) to more severe forms such as persistent diarrhea (lasting 14 days or more) leading to malnutrition, hospitalizations and even death [1,2,5,7–13]. Immunocompromised hosts, e.g., persons with HIV/AIDS and malnourished children in developing countries, are more prone to develop severe clinical illness [1,14]. Fecal shedding of *Cryptosporidium* oocysts can persist for weeks after clinical illness resolves [15,16]. Since *Cryptosporidium* oocysts tolerate chlorination, waterborne outbreaks also occur in industrialized countries [1,3,5].

Recently, the Global Enteric Multicenter Study (GEMS) elucidated the relative importance of *Cryptosporidium* versus many other enteropathogens as a cause of medically-attended diarrhea in young children in developing countries of sub-Saharan Africa (SSA) and South Asia [10], where most young child diarrheal deaths occur. *Cryptosporidium* was the second leading cause (5–15%) of moderate-to-severe diarrhea (MSD) in infants at all 7 GEMS study sites. *Cryptosporidium* remained a leading cause of MSD in toddlers age 12–23 months, ranking third after rotavirus and *Shigella*; 5–9% of all MSD cases in 5 of the 7 sites were attributable to *Cryptosporidium* [10]. *Cryptosporidium*-associated MSD negatively impacted linear growth and significantly increased the risk of death in toddlers [10]. A follow-on study, GEMS-1A, investigated *Cryptosporidium* in association with less-severe diarrhea (LSD) over a 1-year period in 6 of 7 GEMS sites; the LSD cases enrolled in GEMS-1A, like the MSD cases enrolled in GEMS, were pediatric patients who were brought to health care facilities.

We extrapolated GEMS site-specific burdens of *Cryptosporidium*-associated MSD and LSD in children age <24 months to estimate *Cryptosporidium*-associated diarrhea burdens for the entire SSA region (except the Republic of South Africa) and the India/Pakistan/Bangladesh/Nepal/Afghanistan (I/P/B/N/A) region of South Asia, where ~80% of global young child deaths due to diarrheal disease occur [17,18].

## Methods

### Study design and population

GEMS was a prospective matched case-control study conducted for 36 months at 7 sites where demographic surveillance systems (DSS) regularly updated censused populations. Sites included: Basse, The Gambia; Bamako, Mali; Manhiça, Mozambique; Siaya County, Kenya; Kolkata, India; Mirzapur, Bangladesh; and Bin Qasim Town, Pakistan. The published rationale [19], working assumptions [20], epidemiological [21], laboratory [22], and statistical methods [23] of GEMS are summarized below.

The GEMS sampling frame comprised children age <60 months residing within each site’s DSS area. Children brought to sentinel health centers (SHCs) serving each DSS were assessed for criteria for MSD (*vide infra*). Every fortnight, 8–9 cases were targeted for enrollment, per age stratum (0–11, 12–23 and 24–59 months), per site. Within 14 days of each case enrolled, we undertook to enroll 1–3 randomly selected age- and sex-matched controls from the same or nearby communities. MSD was defined as a new acute diarrheal episode (≥3 loose stools in the previous 24 hours, occurring after ≥7 diarrhea-free days, and beginning within the previous 7 days), and having some or severe dehydration, initiation of intravenous rehydration based on a clinician’s judgment, visible blood in stools (dysentery), or hospitalization for diarrhea or dysentery. At enrollment, a standardized evaluation, anthropometric measurements, and a stool sample were obtained from cases and controls. A single follow-up home visit was carried out ~60 (range 49–91) days after enrollment, during which the vital status of cases and controls was recorded and anthropometric measurements were made.

GEMS-1A was a 1-year extension in which children with MSD and LSD were enrolled at the SHCs in 6 of 7 GEMS sites, while in Kenya only MSD cases were enrolled. LSD was defined as a new acute diarrhea case seen at SHCs that did not meet the definition of MSD. Data collection methods, including the ~60-day follow-up household visit, were otherwise identical to GEMS.

### Laboratory procedures

Case and control stool samples were tested for numerous enteropathogens [10,22], including *Cryptosporidium*, which was detected using an enzyme immunoassay (EIA) (TechLab, Inc. Blacksburg, VA). A random subset of stool specimens from 3,809 GEMS MSD case-control pairs from across all sites was also tested for various enteropathogens using TaqMan Array Card (TAC)-based real-time polymerase chain reaction (PCR). Briefly, nucleic acid was extracted from stool specimens using the QIAamp Fast Stool DNA Mini kit (Qiagen, Valencia, CA). The TaqMan Array Card methodology compartmentalizes PCR reactions for 48 targets per specimen as previously described [24]. For this project we included primers to amplify the 18S rRNA gene of *Cryptosporidium* species [24] and primers for alleles of the LIB13 locus that differentiates *C*. *hominis* from *C*. *parvum* [25]. The LIB13 locus is not known to be present in other *Cryptosporidium* species, other than a divergent sequence in *C*. *cuniculus* (GU327781). The assay did not amplify genomic DNA from *C*. *meleagridis* isolate TU1867. Specimens with cycle threshold (CT) values ≤40 were considered positive with the species identification assay. For specimens that did not yield a LIB13 result, we performed nested amplification of a longer fragment of the 18S gene rRNA [26], as well as GP60 to try to identify the species [27]. GP60 sequencing was also performed to subtype the available *C*. *parvum* and *C*. *hominis* specimens.

### Estimating the burden attributable to *Cryptosporidium*

**Fig 1:**
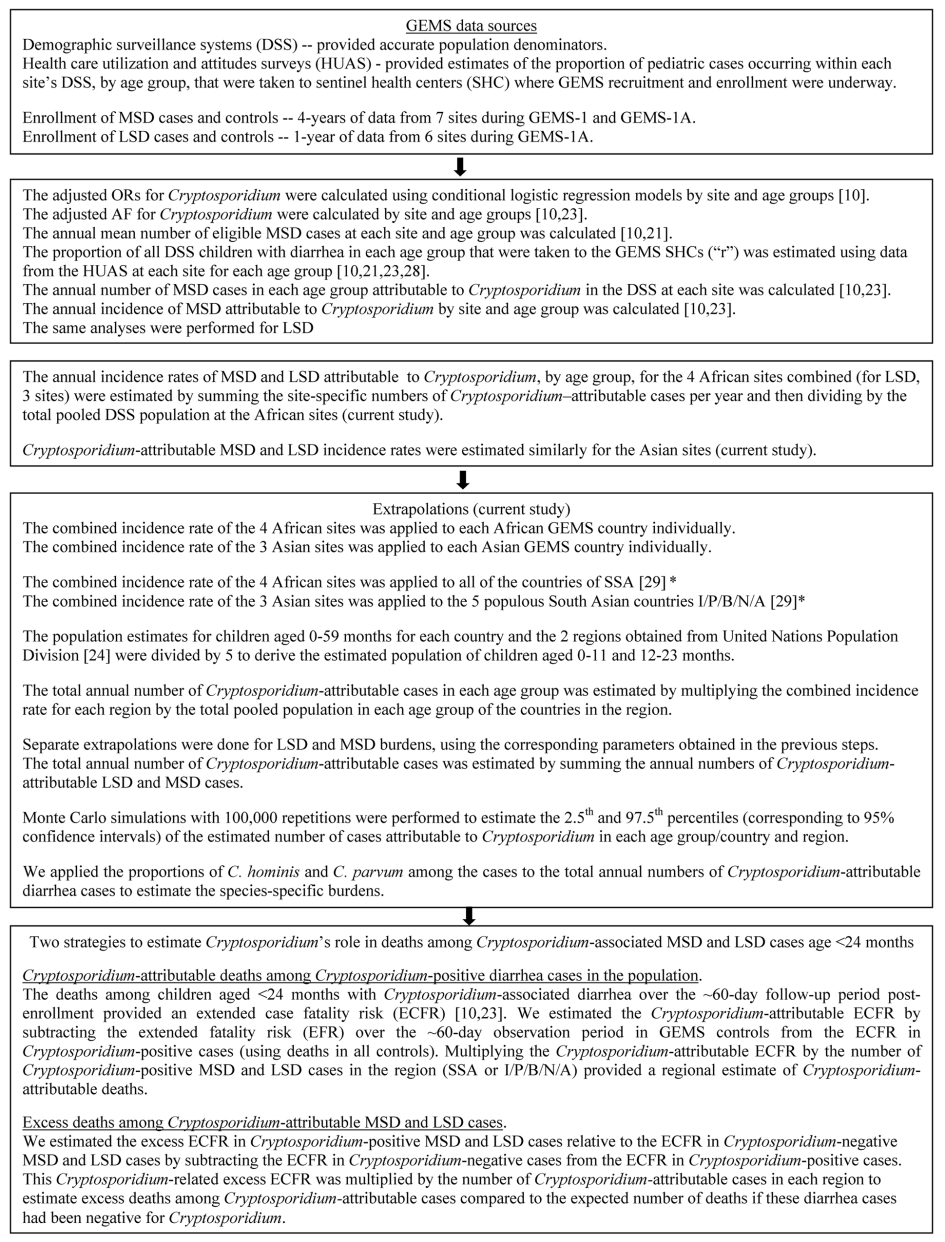
Flow chart of steps and methods used in calculating the burden attributable to *Cryptosporidium* diarrhea. AF: attributable fraction, DSS: Demographic Surveillance systems, GEMS: Global Enteric Multicenter Study, HUAS: Health care utilization and attitudes surveys, region, LSD: less severe diarrhea, MSD: moderate-to-severe diarrhea, OR: odds ratio, SHC: sentinel health centers * I/P/B/N/A: India, Pakistan, Bangladesh, Nepal and Afghanistan countries of South Asia; SSA: sub-Saharan Africa (excluding South Africa).

Since *Cryptosporidium* was incriminated as a cause of MSD and LSD mainly among children aged <24 months [10], disease burden extrapolations focused on infants 0–11 and toddlers 12–23 months of age. Details of the analysis are presented in Fig 1. For each site and age group, pathogen-specific attributable fractions (AFs), weighted according to calendar time and presence or absence of dysentery and adjusted for the presence of other pathogens, and annual attributable incidence (AI) rates for MSD during the 3 years of GEMS have been reported [10]. Employing similar methodology [10,23,28], GEMS-1A data were used to estimate *Cryptosporidium*-attributable incidence of LSD, for site/age groups in which *Cryptosporidium* was associated with LSD with P<0.1 after adjustment for other pathogens. Data from GEMS and GEMS-1A were used to estimate odds ratios (ORs) for *Cryptosporidium* and MSD by 6-month age interval for children aged 0–23 months; weighting by time or presence of dysentery should have little effect on associations with *Cryptosporidium*, and it was not employed in this analysis.

*Cryptosporidium*-specific AFs and AI rates, healthcare utilization rates for MSD and LSD, along with population estimates for the sites, were used to calculate overall *Cryptosporidium*-specific MSD and LSD AI rates separately for the 4 African sites (3 sites for LSD) and 3 Asian sites. These AI rates were extrapolated to the countries where GEMS sites were located, the 51 countries of the SSA region (excluding Republic of South Africa), and the India/Pakistan/Bangladesh/Nepal/Afghanistan (I/P/B/N/A) region of South Asia. For each country or region, the *Cryptosporidium* AI rate was multiplied by the total population (per United Nations estimates) [29] to generate national and region-wide estimates of annual *Cryptosporidium*-attributable MSD and LSD cases. Since GEMS and GEMS-1A were conducted over 5 calendar years, we used the average UN estimated population size (for each GEMS country and region) of children 0–4 years of age during 2005–2010; we divided by 5 to estimate the number of children aged 0–11 and aged 12–23 months. To estimate 95% confidence intervals (CIs), we took the 2.5^th^ and 97.5^th^ percentiles of the number of *Cryptosporidium*-attributable diarrhea cases from 100,000 Monte Carlo simulations, assuming normal distributions for relevant parameters, with standard deviations estimated from Taylor series approximations. The proportions of *C*. *hominis* and *C*. *parvum* among the subset of cases (by PCR) were multiplied by the total number of *Cryptosporidium*-attributable diarrhea cases to estimate the species-specific attributable burdens.

### Two strategies to estimate deaths among *Cryptosporidium*-associated diarrhea cases

The number of deaths among children aged <24 months with *Cryptosporidium*-associated diarrhea over the ~60-day follow-up period following enrollment provided an extended case fatality risk (ECFR) [10,21]. However, because GEMS and GEMS-1A were conducted in populations with high or moderate <5 years mortality, and given numerous risk factors for death among children with MSD or LSD, some proportion of deaths among *Cryptosporidium*-associated diarrhea cases would have occurred unrelated to *Cryptosporidium* infection. Accordingly, we utilized two different analytical strategies to estimate more specifically the role of *Cryptosporidium* in deaths of children with *Cryptosporidium*-associated diarrheal illness.

### *Cryptosporidium*-attributable deaths among *Cryptosporidium*-positive diarrhea cases in the population age <24 months

First, we estimated the *Cryptosporidium*-attributable ECFR by subtracting the extended fatality risk (EFR) over the ~60-day observation period in GEMS/GEMS-1A controls from the ECFR in *Cryptosporidium*-positive cases. We used deaths in all controls, because the numbers of deaths among matched controls of *Cryptosporidium*-positive cases were very small. Multiplying the *Cryptosporidium*-attributable ECFR by the estimated number of *Cryptosporidium*-positive MSD and LSD cases in the region (SSA or I/P/B/N/A) provides a regional estimate of *Cryptosporidium*-attributable deaths.

### Excess deaths among *Cryptosporidium*-attributable MSD and LSD cases

We estimated the excess ECFR in *Cryptosporidium*-positive MSD and LSD cases relative to the ECFR in *Cryptosporidium*-negative cases by subtracting the ECFR in *Cryptosporidium*-negative cases from the ECFR in *Cryptosporidium*-positive cases. This excess risk represents the contribution of *Cryptosporidium* to death risk beyond both the background risk of death in the general pediatric population and that of diarrhea patients. The excess ECFR was then multiplied by the number of *Cryptosporidium*-attributable cases in each region, to estimate excess deaths among *Cryptosporidium*-attributable cases compared to the expected number of deaths if these cases had been *Cryptosporidium*-negative.

Estimates of *Cryptosporidium*-related deaths for the combined age group 0–23 months were calculated separately for MSD and LSD for the SSA region, given the much higher ECFR in children with MSD; deaths were estimated for MSD and LSD combined for the I/P/B/N/A region. Two-sided 95% CIs for differences in death risks were estimated by the Miettinen and Nurminen likelihood score method [30]. CIs for numbers of deaths were estimated assuming normal distributions for numbers of deaths, with variances estimated from Taylor series approximations.

Statistical significance was defined as a two-sided P-value <0.05. Analyses were performed using SAS version 9, IBM SPSS version 22, and NCSS 8.

### Ethical approval

The study protocol was approved by ethics committees at the University of Maryland, Baltimore and at each field site [21]. Parents/caregivers of participants provided written informed consent, and a witnessed consent was obtained for illiterate parents/caretakers.

## Results

### Patterns of infection and diarrheal illness associated with *Cryptosporidium*, by age

Among 15,284 cases (12,110 MSD and 3,174 LSD) and 21,527 matched controls from GEMS-1 and GEMS-1A, *Cryptosporidium* data were missing for 11 cases (0.07%) and 10 controls (0.0046%) and these participants were excluded from analyses. Among the total 15,284 MSD and LSD cases, six (0.039%) had four matched controls rather than a maximum of three; these six deviations occurred in one African site during GEMS-1. The six extra matched controls were not censured from the dataset. Overall, *Cryptosporidium* was detected in stools from 1632 cases (10.7%) and 1184 controls (5.5%) (P<0.001); positivity was significantly higher in MSD cases than matched controls in the age groups 0–11 and 12–23 months at all sites, and among the 24–59 month age group in Kenya. *Cryptosporidium* was also significantly more common in LSD cases than controls aged 0–11 and 12–23 months in Gambia and India, while in Mali and Mozambique this was found only in the toddler age group and in Pakistan only in infants and in children age 24–59 months (Table 1). Adjusted attributable incidence rates of *Cryptosporidium* LSD by site and age group are shown in Table 2.

**Table 1:** *Cryptosporidium* positivity (by EIA) in cases and controls by age, site, and severity of diarrhea.

	Basse, The Gambia		Bamako, Mali		Manhiça, Mozambique		Siaya County, Kenya		Kolkata, India		Mirzapur, Bangladesh		Karachi, Pakistan	
**MSD**	**Cases**	**Controls**	**Cases**	**Controls**	**Cases**	**Controls**	**Cases**	**Controls**	**Cases**	**Controls**	**Cases**	**Controls**	**Cases**	**Controls**
**Total number (age 0–11 months)**	520	783	961	961	439	883	829	896	878	892	672	1122	788	788
***Cryptosporidium* (%)**	*16*.*0*	*6*.3*	*16*.*6*	*6*.9*	*19*.*8*	*8*.8*	*14*.*4*	*5*.8*	*15*.*3*	*6*.7*	*8*.*2*	*3*.4*	*14*.*1*	*9*.1*
**Total number (age 12–23 months)**	609	894	911	924	237	517	491	808	752	778	579	967	512	902
***Cryptosporidium* (%)**	*12*.*3*	*5*.0*	*9*.*4*	*6*.9*	*16*.*5*	*9*.9*	*11*.*0*	*4*.8*	*14*.*4*	*8*.2*	*6*.*0*	*3*.3*	*10*.*9*	*5*.7*
**Total number (age 24–59 months)**	243	503	845	863	137	279	458	744	477	923	463	1111	298	745
***Cryptosporidium* (%)**	*3*.*7*	*2*.*2*	*3*.*9*	*3*.*0*	*6*.*6*	*6*.*8*	*4*.*8*	*1*.7*	*9*.*6*	*12*.*0*	*4*.*5*	*5*.*3*	*4*.*7*	*3*.*2*
**LSD**	**Cases**	**Controls**	**Cases**	**Controls**	**Cases**	**Controls**	**Cases**	**Controls**	**Cases**	**Controls**	**Cases**	**Controls**	**Cases**	**Controls**
**Total number (age 0–11 months)**	220	259	236	236	155	154	NA	NA	213	213	183	366	227	228
***Cryptosporidium* (%)**	*13*.*6*	*5*.4*	*9*.*3*	*6*.*8*	*16*.*1*	*10*.*4*			*7*.*5*	*1*.9*	*4*.*4*	*1*.*9*	*9*.*3*	*3*.1*
**Total number (age 12–23 months)**	202	273	226	227	175	175	NA	NA	180	194	148	296	171	309
***Cryptosporidium* (%)**	*11*.*9*	*4*.8*	*11*.*1*	*5*.7*	*15*.*4*	*6*.3*			*5*.*6*	*1*.0*	*3*.*4*	*2*.*0*	*10*.*5*	*6*.*8*
**Total number (age 24–59 months)**	135	250	230	230	101	101	NA	NA	181	187	83	248	108	288
***Cryptosporidium* (%)**	*7*.*4*	*3*.*6*	*4*.*3*	*2*.*6*	*9*.*9*	*3*.*0*			*3*.*3*	*1*.*6*	*3*.*6*	*2*.*0*	*5*.*6*	*1*.4*

* P < 0.05 for the difference between cases and controls, as obtained in unadjusted conditional logistic regression models.

MSD: moderate-to-severe diarrhea, LSD: less severe diarrhea, EIA: enzyme immune assay, NA: not applicable, the Kenyan site did not enroll LSD cases in the GEMS-1A component

**Table 2:** Adjusted attributable incidence (per 100 child-years) and 95% confidence intervals (CIs) of *Cryptosporidium*-attributable LSD, by site and age group*.

Site/ age group (months)	Adjusted attributable incidence rate per 100 child-years (95% CI)
**Gambia**	
0–11	4.58 (-0.01–9.53)
12–23	3.49 (-0.01–6.98)
24–59	0.39 (-0.15–0.92)
**Mali**	
0–11	-
12–23	3.92 (-3.10–10.95)
24–59	-
**Mozambique**	
0–11	5.36 (-4.90–15.62)
12–23	5.47 (-1.32–12.26)
24–59	-
**India**	
0–11	4.73 (0.61–8.86)
12–23	3.43 (-0.78–7.64)
24–59	-
**Bangladesh**	
0–11	-
12–23	-
24–59	-
**Pakistan**	
0–11	8.46 (-0.03–16.95)
12–23	10.81 (-1.19–22.81)
24–59	0.93 (-0.24–2.09)

* Weighted adjusted attributable incidence rates were calculated only for site/age groups in which *Cryptosporidium* was associated with LSD with P<0.1 in multivariable conditional logistic regression models that adjusted for the presence of other enteric pathogens.

Note—LSD cases were not enrolled in Kenya during GEMS-1A

When MSD data were examined in narrower age groups, we found significant positive associations between *Cryptosporidium* and MSD at 4 sites (Mali, Mozambique, Kenya, India) in the first 5 months of life. ORs were higher at age 6–11 months, except in Mali. A significant OR between *Cryptosporidium* and MSD was observed in Mali, Mozambique, Kenya and India at ages 0–17 months. In Gambia and Pakistan, this association was significant from 6–23 months of age and in Bangladesh only at age 6–11 months. Adjusted AFs increased from age 0–5 months to age 6–11 months and were highest at age 6–11 months, except in Pakistan (Table 3).

**Table 3:** Association between *Cryptosporidium* (by EIA) and MSD from age 0–23 months by site during four years of surveillance: matched unadjusted and adjusted odds ratios (ORs) and adjusted attributable fractions (AF) with 95% confidence intervals (CIs)*.

Site and age group (months)	Number of MSD cases	Number (%) of cases positive for *Cryptosporidium*	Number of controls	Number (%) of controls positive for *Cryptosporidium*	Unadjusted OR (95% CI)	P^1^	Adjusted OR (95% CI)	P^2^	Adjusted AF (95% CI)
**Gambia**									
<6	86	7 (8.1)	123	6 (4.9)	1.60 (0.50–5.18)	0.43	1.99 (0.58–6.82)	0.28	4.0 (-3.0–11.1)
6–11	434	76 (17.5)	660	43 (6.5)	4.00 (2.47–6.47)	<0.0001	4.41 (2.65–7.35)	<0.0001	13.5 (9.3–17.8)
12–17	338	45 (13.3)	478	25 (5.2)	2.87 (1.62–5.08)	0.0003	2.61 (1.38–4.92)	0.003	8.2 (3.5–13.0)
18–23	271	30 (11.1)	416	20 (4.8)	2.48 (1.34–4.61)	0.004	3.23 (1.59–6.53)	0.001	7.6 (3.1–12.2)
**Mali**									
<6	230	26 (11.3)	230	4 (1.7)	7.29 (2.34–22.71)	0.0006	6.59 (2.07–20.95)	0.001	9.6 (5.1–14.1)
6–11	731	134 (18.3)	731	62 (8.5)	2.58 (1.83–3.64)	<0.0001	3.76 (2.48–5.70) (*Giardia* absent)^3^ 1.24 (0.56–2.77) (*Giardia* present)^3^	<0.0001 0.59	12.3 (8.7–15.8)
12–17	527	60 (11.4)	533	40 (7.5)	1.70 (1.07–2.71)	0.025	1.81 (1.10–2.98)	0.019	5.1 (1.1–9.1)
18–23	384	26 (6.8)	391	24 (6.1)	1.10 (0.60–2.01)	0.76	1.06 (0.56–2.02)	0.85	0.4 (-3.9–4.7)
**Mozambique**									
<6	153	22 (14.4)	293	26 (8.9)	2.29 (1.18–4.43)	0.014	2.41 (1.17–4.98)	0.018	8.4 (2.0–14.8)
6–11	286	65 (22.7)	589	52 (8.8)	3.67 (2.32–5.83)	<0.0001	5.71 (3.32–9.82)	<0.0001	18.7 (13.2–24.3)
12–17	155	28 (18.1)	330	32 (9.7)	2.45 (1.35–4.42)	0.003	2.30 (1.19–4.44)	0.013	10.2 (2.7–17.8)
18–23	82	11 (13.4)	187	19 (10.2)	1.70 (0.74–3.91)	0.21	1.80 (0.70–4.63)	0.22	6.0 (-4.0–16.0)
**Kenya**									
<6	306	37 (12.1)	329	17 (5.2)	2.44 (1.33–4.46)	0.004	3.09 (1.55–6.16)	0.001	8.2 (3.7–12.7)
6–11	523	82 (15.7)	567	35 (6.2)	2.79 (1.81–4.31)	<0.0001	3.31 (2.08–5.27)	<0.0001	10.9 (7.2–14.7)
12–17	313	38 (12.1)	516	26 (5.0)	3.06 (1.75–5.37)	<0.0001	3.75 (2.02–6.95)	<0.0001	8.9 (4.7–13.1)
18–23	178	16 (9.0)	292	13 (4.5)	2.29 (1.02–5.17)	0.045	2.43 (1.01–5.86)	0.048	5.3 (-0.1–10.7)
**India**									
<6	298	45 (15.1)	301	22 (7.3)	2.35 (1.33–4.13)	0.003	2.44 (1.34–4.44)	0.003	8.9 (3.5–14.3)
6–11	580	89 (15.3)	591	38 (6.4)	2.97 (1.91–4.63)	<0.0001	3.22 (1.90–5.47)	<0.0001	10.6 (6.6–14.6)
12–17	458	65 (14.2)	473	33 (7.0)	2.20 (1.40–3.47)	0.0007	2.19 (1.23–3.87)	0.007	7.7 (2.9–12.5)
18–23	294	43 (14.6)	305	31 (10.2)	1.59 (0.91–2.77)	0.11	2.09 (0.99–4.40)	0.054	7.6 (0.9–14.3)
**Bangladesh**									
<6	165	7 (4.2)	272	10 (3.7)	1.21 (0.44–3.33)	0.70	0.92 (0.31–2.76)	0.88	-0.4 (-4.5–3.8)
6–11	507	48 (9.5)	850	28 (3.3)	3.06 (1.91–4.93)	<0.0001	4.39 (2.15–8.96) (*C*. *jejuni* absent)^4^ 0.79 (0.24–2.59) (*C*. *jejuni* present)^4^	<0.00010.70	5.7 (2.3–9.1)
12–17	341	19 (5.6)	569	18 (3.2)	1.97 (0.99–3.94)	0.054	2.23 (0.76–6.54)	0.14	3.1 (-1.1–7.2)
18–23	238	16 (6.7)	398	14 (3.5)	1.77 (0.84–3.73)	0.13	0.65 (0.15–2.78)	0.56	-3.6 (-16.1–9.0)
**Pakistan**									
<6	315	35 (11.1)	315	27 (8.6)	1.33 (0.78–2.25)	0.30	1.43 (0.77–2.65)	0.26	3.3 (-1.8–8.5)
6–11	473	76 (16.1)	473	45 (9.5)	1.81 (1.22–2.68)	0.003	2.91 (1.71–4.93) (*Aeromonas* absent)^5^ 0.64 (0.16–2.60) (*Aeromonas* present)^5^	<0.0001 0.54	8.5 (3.3–13.7)
12–17	314	31 (9.9)	550	33 (6.0)	1.81 (1.04–3.14)	0.036	2.32 (1.24–4.34)	0.009	5.6 (1.6–9.6)
18–23	198	25 (12.6)	352	18 (5.1)	2.48 (1.28–4.79)	0.007	3.09 (1.47–6.50)	0.003	8.5 (3.3–13.8)

* Table 3 includes data only from study children with *Cryptosporidium* results. EIA: enzyme immunoassay

^1^ These p-values were obtained from unadjusted conditional logistic regression analysis.

^2^ These p-values were obtained from adjusted conditional logistic regression analysis.

^3^ There is an interaction (P < 0.1) between *Cryptosporidium* and *Giardia* for Mali at age 6–11 months.

^4^ There is an interaction (P < 0.1) between *Cryptosporidium* and *C*. *jejuni* for Bangladesh at age 6–11 months.

^5^ There is an interaction (P < 0.1) between *Cryptosporidium* and *Aeromonas* for Pakistan at age 6–11 months.

### *Cryptosporidium* species

We identified *Cryptosporidium* species by PCR testing for 18S and Lib13 targets in a random subset of 3,809 case/control pairs. Samples with unresolved species by Lib13 (which only differentiates *C*. *hominis* from *C*. *parvum*) had species investigated by 18S and GP60 assays, as described in the Methods. This revealed 338 EIA+/PCR+ cases and 157 EIA+/PCR+ controls. Among the 338 samples from *Cryptosporidium*-positive MSD cases, 333 were suitable for further testing, of which 259 (77.8%), 33 (9.9%), 4 (1.2%), and 2 (0.6%), respectively, were positive for *C*. *hominis*, *C*. *parvum*, both *C*. *hominis* and *C*. *parvum* and *C*. *meleagridis*; the species of 35 (10.5%) specimens remained undetermined. Corresponding percentages in controls were 68.2%, 8.9%, 0.6%, 0.6%, and 21.0%. The species of one control sample (0.6%) was identified as *C*. *canis*. GP60 subtypes were identified on 71 EIA+/PCR+ specimens including 32 *C*. *hominis*, 37 *C*. *parvum*, and 2 *C*. *meleagridis*. Of 37 *C*. *parvum* infections, 34 (91.9%) were anthroponotic strains: 21 were IIc (19 A5G3 and 2 A4G3); 13 were IIe (1 IIeA6G1, 2 IIeA7G1, 7 IIeA10G1, 2 IIeA11, 1 IIeA15); all three non-anthroponotic strains were IIdA15G1. These derived from Mali (n = 13), Kenya (n = 9), Mozambique (n = 5), Pakistan (n = 7 including the 3 non-anthroponotic types), Bangladesh (n = 2), and Gambia (n = 1). Samples from Kenya and Mozambique were mostly IIcA5G3 (12/14). Mali’s strains were diverse; containing 10/11 IIe strains as well as 3 IIcA5G3. The *C*. *hominis* subtypes included Ia (1 A18R2, 1 A19R2, 1 A23R2, 1 A24G1R2, 2 A25R2, 1 A26R2), Ib (3 A9G3, 8 A13G3), Id (1A14), Ie (exclusively 10 A11G3T3), and If (2 A14G1). Both *C*. *meleagridis* were subtyped as IIIdA6R1.

### Disease burden

The *Cryptosporidium*-attributable MSD incidence was estimated to be 3.48 (95% CI, 2.27–4.67) and 3.18 (95% CI, 1.85–4.52) per 100 child-years in the African and Asian sites, respectively, in the 0–11 months age group. The respective incidences for toddlers aged 12–23 months were 1.41 (95% CI, 0.73–2.08) and 1.36 (95% CI, 0.66–2.05) per 100 child-years. Corresponding *Cryptosporidium*-attributable LSD incidence rates were 2.52 (95% CI, 0.33–5.01) and 4.88 (95% CI, 0.82–8.92) in infants and 4.04 (95% CI, 0.56–7.51) and 4.71 (95% CI, 0.24–9.18) in toddlers, per 100 child-years.

Applying these incidence rates to the pediatric population age <2 years in the SSA and I/P/B/N/A regions, respectively, yielded annual estimated *Cryptosporidium* MSD burdens of ~1.2 million and ~1.5 million cases. The total number of LSD and MSD cases was estimated to be 2.9 million in this age group in SSA and 4.7 million in the populous I/P/B/N/A region (Table 4). The proportions of cases due to *C*. *hominis* and *C*. *parvum* in the subset tested for species were multiplied by the total estimated number of *Cryptosporidium*-attributable diarrhea cases in both regions (~7.6 million), yielding estimates of 5.9 million *C*. *hominis*, 0.76 million *C*. *parvum* and 90,000 thousand co-infected (*C*. *hominis* plus *C*. *parvum*) cases in children aged <2 years.

**Table 4:** Estimated number (in thousands) of diarrhea cases attributable to *Cryptosporidium* by age, country and region^§^.

Syndrome/age (months)	Gambia	Mali	Mozambique	Kenya**	India	Bangladesh	Pakistan	Sub-Saharan Africa^†^	South Asia *
**MSD**									
Age 0–11									
Number (2.5^th^-97.5^th^ percentile)	2.0 (1.4–2.9)	17.2 (12.0–25.1)	28.5 (19.9–41.5)	44.4 (31.1–64.8)	784.5 (518.0–1,263.3)	100.9 (66.6–162.6)	129.6 (85.5–209.0)	880.5 (616.3–1,283.3)	1,068.0 (705.5–1,719.3)
Age 12–23									
Number (2.5^th^-97.5^th^ percentile)	0.8 (0.5–1.4)	7.0 (4.1–11.9)	11.6 (6.8–19.7)	18.8 (10.6–30.8)	334.7 (187.0–576.0)	43.0 (24.0–74.1)	55.3 (30.8–95.2)	357.2 (210.2–610.2)	455.7 (254.6–784.1)
Age 0–23									
Number (2.5^th^-97.5^th^ percentile)	2.8 (2.1–3.8)	24.8 (17.7–32.7)	41.1 (29.5–54.0)	64.2 (46.0–84.3)	1,119.2 (791.7–1,596.6)	143.9 (101.7–205.5)	184.8 (130.6–264.0)	1,237.7 (910.5–1,671.1)	1,523.8 (1,078.1–2,172.9)
LSD**									
Age 0–11									
Number (2.5^th^-97.5^th^ percentile)	1.5 (0.2–4.0)	12.5 (1.4–34.4)	20.7 (2.3–57.0)	32.3 (3.6–88.9)	1,201.7 (314.2–2,469.2)	154.5 (40.3–317.4)	198.5 (51.7–407.6)	639.3 (71.9–1,761.9)	1,636.1 (427.4–3,361.4)
Age 12–23									
Number (2.5^th^-97.5^th^ percentile)	2.3 (-1.5–9.5)	20.0 (-12.6–81.8)	33.1 (-20.7–135.7)	51.6 (-32.4–211.6)	1,162.1 (219.9–2,687.4)	149.4 (28.3–345.6)	191.9 (36.3–443.8)	1,023.3 (-642.1–4,192.8)	1,582.1 (299.5–3,658.1)
Age 0–23									
Number (2.5^th^-97.5^th^ percentile)	3.8 (-0.7–12.4)	32.5 (-6.4–106.6)	53.8 (-10.6–176.4)	83.9 (-16.6–275.1)	2,363.8 (1,028.5–4,301.3)	303.9 (132.2–553.0)	390.4 (169.7–710.8)	1,662.6 (-329.0–5,451.8)	3,218.2 (1,400.5–5,855.6)
**MSD & LSD** **									
Age 0–11									
Number (2.5^th^-97.5^th^ percentile)	3.5 (1.8–6.0)	29.7 (15.8–52.0)	49.2 (26.3–86.0)	76.7 (40.9–134.1)	1,986.3 (1,066.4–3,334.7)	255.4 (137.1–428.8)	328.0 (176.0–551.0)	1,519.8 (811.0–2,657.7)	2,704.1 (1,451.9–4,540.0)
Age 12–23									
Number (2.5^th^-97.5^th^ percentile)	3.1 (-1.0–10.2)	26.9 (-8.2–87.5)	44.6 (-13.6–144.9)	69.7 (-21.3–226.1)	1,496.8 (545.5–3,044.6)	192.5 (70.1–391.4)	247.2 (90.1–502.9)	1,380.5 (-421.6–4,479.7)	2,037.8 (742.6–4,147.8)
Age 0–23									
Number (2.5^th^-97.5^th^ percentile)	6.6 (1.8–15.0)	56.6 (15.6–128.5)	93.8 (25.9–212.4)	146.4 (40.4–331.8)	3,483.1 (2,118.7–5,481.1)	447.8 (271.5–705.3)	575.2 (348.6–906.1)	2,900.3 (800.6–6,575.0)	4,741.9 (2,874.8–7,462.5)

^†^ Countries and areas included in the extrapolations to sub-Saharan Africa region: Angola, Benin, Botswana, Burkina Faso, Burundi, Cameroon, Cape Verde, Central African Republic, Chad, Comoros, Congo, Côte d'Ivoire, Democratic Republic of the Congo, Djibouti, Equatorial Guinea, Eritrea, Ethiopia, Gabon, Gambia, Ghana, Guinea, Guinea-Bissau, Kenya, Lesotho, Liberia, Madagascar, Malawi, Mali, Mauritania, Mauritius, Mayotte, Mozambique, Namibia, Niger, Nigeria, Réunion, Rwanda, Saint Helena, São Tomé and Príncipe, Senegal, Seychelles, Sierra Leone, Somalia, Sudan, Swaziland, Togo, Uganda, United Republic of Tanzania, Zambia, and Zimbabwe [29].

* Extrapolation was made to the most populous countries in South Asia: India, Bangladesh, Pakistan, Afghanistan and Nepal [29].

^§^ Data presented are estimated absolute numbers of cases attributable to *Cryptosporidium*, in parentheses are the 2.5^th^ and 97.5^th^ percentiles (corresponding to 95% confidence intervals), which were estimated by Monte Carlo simulations

** Extrapolation of the combined incidence rate of the 3 African sites of GEMS-1A (Gambia, Mali, Mozambique) was made to the population of Kenya.

### *Cryptosporidium*-attributable deaths among *Cryptosporidium*-positive diarrhea cases in the population and all diarrhea-attributable deaths

Subtracting the EFR among all controls of MSD cases aged <24 months at African sites (37/6258, 0.6%) from the ECFR among *Cryptosporidium*-positive MSD cases (41/643, 6.4%) (Table 5) yielded a *Cryptosporidium*-attributable ECFR of 5.8% (95% CI, 4.4%–7.6%). Similarly, subtracting the EFR in controls of LSD cases aged <24 months at African sites (4/1261, 0.3%) from the ECFR among *Cryptosporidium*-positive LSD cases (1/139, 0.7%) produced an estimated *Cryptosporidium*-attributable ECFR of 0.4% (95% CI, -0.4%–3.7%). Multiplying these *Cryptosporidium*-attributable ECFRs by the numbers of *Cryptosporidium*-positive cases generated estimates of 107,000 *Cryptosporidium*-attributable MSD deaths (95% CI, 68,200–151,000) and 16,300 *Cryptosporidium*-attributable LSD deaths (95% CI, 0–75,200) in the SSA region. After subtracting the EFR in controls of MSD and LSD cases at Asian sites (6/6758, 0.09%) from the ECFR among *Cryptosporidium*-positive MSD and LSD cases combined (5/523, 1.0%) in the Asian sites and multiplying the resultant *Cryptosporidium*-attributable ECFR of 0.9% (95% CI, 0.4%–1.9%) by all *Cryptosporidium*-positive diarrhea cases, we estimate 78,900 (95% CI, 0–159,000) deaths attributable to *Cryptosporidium*-positive diarrhea in the (I/P/B/N/A) region. Thus, we estimate a total of ~202,000 *Cryptosporidium*-attributable diarrhea deaths in the two regions combined. Using the same methodology, we estimate 455,000 (95% CI, 280,000–630,000) annual diarrhea-attributable deaths in the SSA region and 254,000 (95% CI, 13,900–494,000) in the (I/P/B/N/A) region.

**Table 5:** Extended fatality risk during ~ 60 days following the onset of acute diarrhea among cases and controls aged 0–23 months*.

	African sites			Asian sites		
Group	Total subjects	No. of deaths during ~60 days of follow-up	Percent (95% CI)	Total subjects	No. of deaths during ~60 days of follow-up	Percent (95% CI)
Total MSD cases	4552	169	3.7% (3.2%-4.3%)	3828	26	0.7% (0.4%-1.0%)
*Cryptosporidium*-positive MSD cases	643	41	6.4% (4.6%-8.6%)	451	4	0.9% (0.2%-2.3%)
*Cryptosporidium*-negative MSD cases	3909	128	3.3% (2.7%-3.9%)	3377	22	0.7% (0.4%-1.0%)
All controls of MSD cases	6258	37	0.6% (0.4%-0.8%)	5204	3	0.06% (0.01%-0.2%)
Controls of *Cryptosporidium*-positive MSD cases	928	4	0.4% (0.1%-1.1%)	573	0	0.0% (0.0%-0.6%)
Total LSD cases	1139	6	0.5% (0.2%-1.1%)	1035	5	0.5% (0.2%-1.1%)
*Cryptosporidium*-positive LSD cases	139	1	0.7% (0.1%-3.9%)	72	1	1.4% (0.3%-7.5%)
*Cryptosporidium*-negative LSD cases	1000	5	0.5% (0.2%-1.1%)	963	4	0.4% (0.2%-1.1%)
All controls of LSD cases	1261	4	0.3% (0.1%-0.8%)	1554	3	0.2% (0.07%-0.5%)
Controls of *Cryptosporidium*-positive LSD cases	156	1	0.6% (0.02%-3.5%)	102	0	0.0% (0.0%-3.6%)

* MSD: moderate-to-severe diarrhea (GEMS-1 and GEMS-1A data), LSD: less severe diarrhea (GEMS-1A data). This table includes data only for cases with *Cryptosporidium* results and non-missing data on vital status at follow-up, and their matched controls.

### Excess deaths among *Cryptosporidium*-attributable MSD and LSD cases

Subtracting the ECFR of *Cryptosporidium*-negative MSD cases (3.3%) from the ECFR of *Cryptosporidium*-positive MSD cases (6.4%) (Table 5) and multiplying the result by the number of *Cryptosporidium*-attributable MSD cases in the SSA region indicated that *Cryptosporidium* was responsible for an excess ~38,400 (95% CI, 13,400–68,100) MSD deaths among *Cryptosporidium*-attributable MSD cases. The analogous calculations based on LSD data (Table 5) suggested that *Cryptosporidium* led to ~3,600 (95% CI, -31,600–46,000) excess LSD deaths per year. In Asian sites the estimate of annual excess deaths in children <24 months of age with *Cryptosporidium*-attributable diarrhea was ~16,900 (95% CI, -20,900–61,100). Thus, between the two regions we estimate that *Cryptosporidium* was responsible for ~59,000excess deaths in cases of *Cryptosporidium*-attributable diarrhea in children aged <24 months, compared to the expected deaths in the same number of *Cryptosporidium*-negative cases.

## Discussion

There have been few attempts to estimate the burden of *Cryptosporidium* diarrheal disease in large populations. One exception is the *Cryptosporidium* burden estimate published for India [31]. Others are the global *Cryptosporidium*-associated mortality estimates contained within Global Burden of Disease (GBD) and Child Epidemiology Estimation Group (CHERG) reports [32–34]. Three obstacles have heretofore impeded attempts to estimate region-wide *Cryptosporidium* disease burdens, including: 1) marked heterogeneity of clinical and laboratory methods used in studies of the etiology of pediatric diarrhea; 2) failure to take into account that many children without diarrhea also excrete *Cryptosporidium*; 3) a lack of species-specific data from clinical studies which might guide vaccine development efforts. In this paper, we utilized datasets and laboratory tests that allowed these obstacles to be overcome.

GEMS-1, pursued in representative sites in 7 developing countries, documented *Cryptosporidium* as a leading cause of endemic childhood diarrhea of a severity that brings children to healthcare facilities, particularly during the first 24 months of life [10]. Importantly, GEMS-1 utilized rigorous standardized clinical, epidemiologic and laboratory methods to collect extensive data over several consecutive years in 7 sites. By including matched control children without diarrhea and adjusting for mixed infections with other enteropathogens, GEMS-1 quantified the specific role of *Cryptosporidium* in childhood diarrheal disease beyond the background carriage of *Cryptosporidium* [10,23]. Finally, our results represent the first systematic, multisite, geographically-diverse assessment of the species-specific burden of *Cryptosporidium*-associated pediatric diarrhea, unequivocally corroborating the dominance of *C*. *hominis* [7,35–38] in infants and toddlers at all sites and revealing that 92% of *C*. *parvum* infections were due to recognized anthroponotic subtypes [39–41].

Despite our cautious extrapolation strategy, we found a substantial disease burden of ~7.6 million diarrhea cases annually attributable to *Cryptosporidium*, including ~2.9 million in SSA and ~4.7 million in the I/P/B/N/A region. Our estimated annual number of *Cryptosporidium*-attributable diarrhea cases (3.5 million) among Indian children aged <24 months falls within the lower limit of the burden estimated by Sarkar *et al*. [31].

GEMS-1 [10] and other studies [8,13,42] have demonstrated a negative impact of *Cryptosporidium*-associated diarrhea on linear growth (stunting), a nutritional insult that increases the risk for severe or fatal outcomes [43,44]. The Malnutrition and Enteric Infections (MAL-ED) study prospectively followed birth cohorts in Peru, Brazil, Tanzania, South Africa, Pakistan, Bangladesh, Nepal and India with twice-weekly household visits through age 24 months [45], thereby detecting mostly mild diarrheal episodes typically not observed in healthcare facility-based passive surveillance. Thus, MAL-ED provides data on the etiology of milder diarrheal illness and revealed *Cryptosporidium* to be the fifth most important diarrhea-associated pathogen in the first year of life and seventh most important in the second year of life [45]. Regional burden estimates that we calculated did not incorporate the burden of milder clinical forms of *Cryptosporidium*-associated illness detected by active-surveillance household visits, as in MAL-ED, and thus probably under-estimates total burden.

The two models of the death burden in children with *Cryptosporidium* described in this manuscript demonstrated that among the 4 African sites MSD cases infected with *Cryptosporidium* at enrollment had a significantly increased ECFR during the subsequent ~60-days [10] compared to the risk of death in controls and to the risk of death in *Cryptosporidium*-negative diarrhea cases. ECFR in children with *Cryptosporidium* in the African sites was particularly driven by Mozambique (high HIV prevalence) and rural Gambia (low HIV), suggesting that factors other than HIV infection, such as malnutrition, play a role in *Cryptosporidium*-related deaths in SSA. While overall mortality during the 60-day follow-up was much lower in the Asian sites [10], nevertheless our estimates indicate a substantial *Cryptosporidium*-related death burden because of the enormity of the <2 years population. Our GEMS-based estimates of deaths under 24 months attributable to *Cryptosporidium* diarrhea are greater than the estimates reported by GBD (35,200 deaths) [33] and CHERG (12,000) [34] among children age <5 years. Discrepancies between GBD and CHERG estimates of <5 years diarrheal disease mortality are recognized [18,46,47]. Our estimates of total diarrhea-attributable deaths (455,000 and 254,000 in the SSA and I/P/B/N/A regions, respectively) are somewhat larger than estimates for children <5 years of age for 2011 in a recent review [48]. In large part these differences are likely because the GEMS estimates are uniquely based on follow-up information on deaths among laboratory-diagnosed *Cryptosporidium*-associated diarrhea cases, whereas CHERG and GBD estimates are based on deaths that occur acutely.

Our observational study design does not permit definitive determination of the direct causation between *Cryptosporidium*-positivity and deaths in young children. Nonetheless, the GEMS-based findings corroborate other results from West Africa [9] highlighting *Cryptosporidium* as a very clear strong signal for children at increased risk for death. From a public health perspective, this is sufficient to plan interventions aimed at reducing the risk of death in such high-risk groups.

We observed a general age-specific pattern of *Cryptosporidium* infection and a strong association with MSD, with documentation of exposure in the first few months of life (in both cases and controls), a peak adjusted Attributable Fraction at age 6–11 months and a decrease thereafter (Table 3). We interpret this as reflecting a time-limited (first 5 months of life), passive protection mediated by maternally-transferred serum IgG as well as secretory IgA antibodies and other protective components of breast milk, despite exposure to the pathogen. Over the first two years of life, the prevalence of *Cryptosporidium* positivity in the controls remains impressively static documenting continuing exposure. However, beginning at ~6 months of age, clinical episodes of *Cryptosporidium*-associated diarrheal illness become more common and continue through age 23 months. By ~24 months of age these clinical and subclinical infections appear to induce in most toddlers acquired active immunity against further *Cryptosporidium* clinical illness. Support for this interpretation comes from a cohort study of Bedouin children in Southern Israel, a population under transition [49]. Serum IgG and IgM antibodies to a *Cryptosporidium* oocyst lysate were measured in children ranging from neonates to toddlers age 23 months [49]. High geometric mean titers (GMT) of serum IgG antibodies (of presumed maternal origin) were recorded at birth. GMT then decreased gradually until age 6 months, after which it increased progressively, as did the incidence of diarrheal illness and detection of *Cryptosporidium* in stools [49]. Collectively, these observations can be interpreted as indicating that immunity against *Cryptosporidium* develops following natural exposure to the pathogen. Measurements of serum anti-gp15 antibody and clinical and sub-clinical infections were also monitored in a cohort of infants and toddlers in Vellore, India. Children who lacked anti-gp15 antibodies just before weaning had higher rates of *Cryptosporidium* infections (77%) than seropositive children (59%), although with the relatively small numbers in the cohort the difference did not reach statistical significance (p = 0.076) [50].

Studies of infection-derived immunity in gnotobiotic piglets further support the notion of acquired immunity to *C*. *hominis*, as an initial induced *C*. *hominis* gastroenteritis in piglets significantly protected them against subsequent re-challenge with *C*. *hominis* [51]. Our documentation of the predominance of *C*. *hominis* over *C*. *parvum* as a pathogen for human infants implies that vaccine development research should prioritize protection against this species and against anthroponotic (human host-restricted) subtypes of *C*. *parvum*. The lower incidence of *Cryptosporidium* disease in infants <6 months old provides a window wherein multiple spaced doses of a future vaccine administered to infants may elicit protection for the subsequent increased risk of clinical *Cryptosporidium* disease encountered from age 6 to 23 months.

Our study has three obvious limitations. While we have extensive data on MSD cases from 7 sites over 4 years, we have only 1-year enrollment of LSD cases from 6 sites. Thus, estimates on children with LSD are less robust than for MSD. Second, we generated pooled estimates of disease incidence and mortality for SSA and I/P/B/N/A and assumed each to be representative of those regions. However, until locally-representative, standardized data become available, our approach is warranted. That *Cryptosporidium* appeared important as a pathogen in both urban and rural, high and low HIV settings, provides evidence that broad extrapolation is justified. Third, the species of a small fraction of *Cryptosporidium*-positive specimens remained unresolved because, although they were positive by EIA and PCR, we could not amplify the long fragments of DNA necessary for species-specific sequence determination of samples mostly with lower parasite load (average 18S PCR Ct 30±4 versus 21±5 for speciated samples, Mann-Whitney U test P<0.001). That said, non-*hominis*/non-*parvum* species appeared to be quite rare. As for the species, the anthroponotic IIc and IIe were predominant *C*. *parvum* subtype families in this study with a larger portion of IIe than often appreciated [39]. Similar findings on *C*. *parvum* subtypes have been described in India [36]. Of 32 *C*. *hominis* infections with GP60 typing data, the predominant subtype families were Ia, Ib, and Ie. Ia had more diverse subtypes [40,52], while Ie subtype was exclusively A11G3T3, consistent with previous reports [36,39]. IbA13G3 infections appear to be common in West Africa. We found these in Mali (n = 4, 2 in cases) and Gambia (n = 4, all in cases), consistent with the high proportions previously seen in Ghana [40]. The observation that the *C*. *parvum* parasites associated with MSD of young children in developing countries represent a restricted anthroponotic subset of all *C*. *parvum* is important as it enhances our ability to better understand the epidemiology of cryptosporidiosis and helps direct our vaccine development efforts.

Currently, there is little research to develop *Cryptosporidium* vaccines for humans and only one licensed drug, nitazoxanide, to ameliorate *Cryptosporidium* diarrhea in children [53]. However, nitazoxanide is currently not recommended for use in infants <12 months of age, exhibits little efficacy in HIV-infected hosts and evidence of efficacy from controlled pediatric trials is limited [53]. The sizable case and death burden of *Cryptosporidium* in the SSA and I/P/B/N/A regions where ~80% of global deaths among young children occur calls for governments, global policymakers, and funding agencies to invest in developing new tools (e.g., vaccines) to prevent *Cryptosporidium* diarrheal illness and improved methods to diagnose and treat it, while also advocating increased access to improved sanitation and safe water.
